# Test-Retest Reliability of Homeostatic Plasticity in the Human Primary Motor Cortex

**DOI:** 10.1155/2018/6207508

**Published:** 2018-06-10

**Authors:** Tribikram Thapa, Siobhan M. Schabrun

**Affiliations:** Brain Rehabilitation and Neuroplasticity Unit, School of Science and Health, Western Sydney University, Locked Bag 1797, Penrith, NSW 2751, Australia

## Abstract

Homeostatic plasticity regulates synaptic activity by preventing uncontrolled increases (long-term potentiation) or decreases (long-term depression) in synaptic efficacy. Homeostatic plasticity can be induced and assessed in the human primary motor cortex (M1) using noninvasive brain stimulation. However, the reliability of this methodology has not been investigated. Here, we examined the test-retest reliability of homeostatic plasticity induced and assessed in M1 using noninvasive brain stimulation in ten, right-handed, healthy volunteers on days 0, 2, 7, and 14. Homeostatic plasticity was induced in the left M1 using two blocks of anodal transcranial direct current stimulation (tDCS) applied for 7 min and 5 min, separated by a 3 min interval. To assess homeostatic plasticity, 15 motor-evoked potentials to single-pulse transcranial magnetic stimulation were recorded at baseline, between the two blocks of anodal tDCS, and at 0 min, 10 min, and 20 min follow-up. Test-retest reliability was evaluated using intraclass correlation coefficients (ICCs). Moderate-to-good test-retest reliability was observed for the M1 homeostatic plasticity response at all follow-up time points (0 min, 10 min, and 20 min, ICC range: 0.43–0.67) at intervals up to 2 weeks. The greatest reliability was observed when the homeostatic response was assessed at 10 min follow-up (ICC > 0.61). These data suggest that M1 homeostatic plasticity can be reliably induced and assessed in healthy individuals using two blocks of anodal tDCS at intervals of 48 hours, 7 days, and 2 weeks.

## 1. Introduction

Synaptic plasticity is fundamental to learning and memory in the human brain. However, synaptic plasticity operates via a positive feedback loop and, as a result, has the potential to destabilise neural networks through excessive synaptic strengthening (long-term potentiation-like effects, LTP) or excessive synaptic weakening (long-term depression-like effects, LTD) [1]. To avoid destabilization, LTP-like and LTD-like changes are subject to homeostatic plasticity mechanisms that maintain the neural activity within an optimal physiological range.

Homeostatic plasticity is theorised to rely on the “sliding threshold” rule, such that the threshold for the induction of LTP or LTD is dependent on the activity in the postsynaptic neuron; high postsynaptic activity favors LTD, whereas low postsynaptic activity favors LTP [2–4]. Although early studies investigating homeostatic plasticity occurred in slice preparations in vitro, a growing body of research has used noninvasive brain stimulation to investigate this mechanism in the human cortex [2–8]. Typically, one noninvasive brain stimulation protocol is used to “prime” (or condition) the synaptic effects of a subsequent noninvasive brain stimulation protocol, and LTP-like and LTD-like effects are indexed using transcranial magnetic stimulation (TMS). For example, when a 5 min block of anodal transcranial direct current stimulation (tDCS) is preceded at a short interval (3 min) by an additional 7 min block of anodal tDCS, the LTP-like (facilitatory) effect of anodal tDCS on the primary motor cortex (M1) is reversed toward LTD (observed as a reduction in corticomotor excitability to TMS) [9]. Similarly, the preconditioning of a 1 Hz repetitive transcranial magnetic stimulation (rTMS) paradigm (that has no overt effect on corticomotor excitability when applied alone) with anodal tDCS produces LTD-like (inhibitory) effects, whereas preconditioning with cathodal tDCS produces LTP-like (facilitatory) effects [10].

Noninvasive brain stimulation has been used to evaluate homeostatic plasticity in M1 in pathological conditions including focal hand dystonia, migraine, and chronic pain [11–14]. These studies demonstrate the impaired homeostatic control in these populations such that the threshold for synaptic plasticity fails to favor the induction of LTD when postsynaptic activity is high. For instance, in individuals with focal hand dystonia, a single block of anodal tDCS increases the corticomotor excitability consistent with the response observed in healthy controls. However, the application of a subsequent block of 1 Hz rTMS fails to reverse the corticomotor excitability toward LTD in this population [15]. Additional studies have provided evidence of paradoxical facilitation in both the visual cortex and M1 of individuals with migraine, observed as an increase in visual cortex and M1 excitability in response to 1 Hz rTMS (in contrast to a reduction in the excitability of both cortices in healthy controls) [16, 17].

Studies comparing M1 homeostatic plasticity between healthy individuals and those with pathology have been limited to cross-sectional designs, despite conditions such as migraine and low back pain being cyclical in nature [12, 14]. To allow the longitudinal evaluation of homeostatic plasticity, as well as the detailed evaluation of the relationship between impaired homeostatic plasticity and symptom status, it is necessary to determine whether homeostatic plasticity can be reliably induced and assessed over time. To our knowledge, no study has investigated the reliability of M1 homeostatic plasticity. Here we aimed to determine the test-retest reliability of M1 homeostatic plasticity, induced and assessed using noninvasive brain stimulation, at intervals of 48 hours, 7 days, and 2 weeks.

## 2. Methods and Materials

### 2.1. Subjects

As no previous multiday studies of homeostatic plasticity exist, a sample size calculation was performed using best available data of MEP amplitudes recorded from healthy individuals at 0, 10, and 20 minutes following an identical double tDCS protocol used to induce and assess homeostatic plasticity in M1 (effect size estimates of 0.4, alpha of 0.05, and power of 0.8) [14]. Using these parameters, ten participants were required to evaluate the test-retest reliability of noninvasive brain stimulation to induce and assess M1 homeostatic plasticity at intervals of 48 hours, 7 days, and 2 weeks. Accordingly, ten right-handed, healthy volunteers (mean ± standard deviation age: 23 ± 5 years, 5 males) were recruited. Handedness was assessed using the Edinburgh handedness questionnaire [18]. All participants were required to meet inclusion criteria as per transcranial magnetic stimulation (TMS) safety guidelines (i.e., no history of epilepsy, absence of metal implants in the skull) [19]. Individuals with a history of neurological, musculoskeletal, upper limb or psychiatric conditions were excluded. A verbal and written description of the experimental procedures was provided to all participants. Written, informed consent was obtained before testing. The study was approved by the institutional Human Research Ethics Committee (approval number: H10184) and performed in accordance with the Declaration of Helsinki.

### 2.2. Experimental Protocol

**Figure 1:**
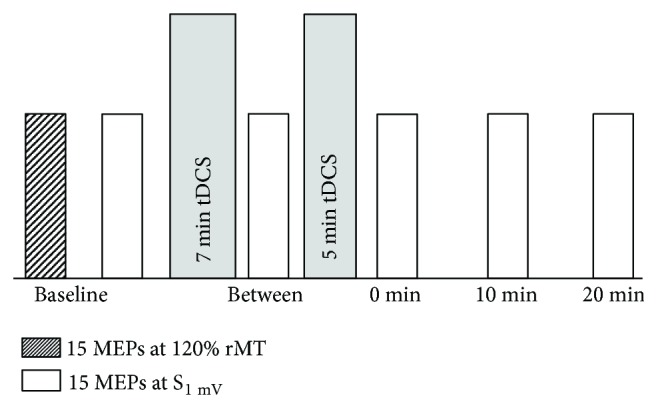
Experimental protocol for days 0, 2, 7, and 14. The corticomotor excitability was assessed at the beginning of each test session using 15 motor-evoked potentials (MEPs) recorded at 120% of resting motor threshold. To ensure a consistent level of baseline corticomotor excitability across subjects prior to the induction of plasticity, further 15 MEPs were recorded at an intensity sufficient to elicit an average MEP of 1 mV peak-to-peak amplitude (S_1mV_) immediately before the first block of 7 min anodal transcranial direct current stimulation (tDCS). This intensity was kept consistent for the remainder of the test session. Plasticity was induced using a 7 min block of anodal tDCS, followed by a second 5 min block of anodal tDCS, separated by a 3 min rest period. Fifteen MEPs were recorded at S_1mV_ between the two blocks of anodal tDCS, and at 0 min, 10 min, and 20 min follow-ups.

Based on intervals used in previous TMS reliability studies [20], corticomotor excitability was assessed, and plasticity was induced in M1, on day 0, 2, 7, and 14. Participants were seated comfortably with their right hand and arm at rest for each test session. To evaluate the change in corticomotor excitability across days, 15 motor-evoked potentials (MEPs) to single-pulse transcranial magnetic stimulation (TMS) were recorded at 120% of resting motor threshold (rMT) at the beginning of each test session. To account for any potential changes in the corticomotor excitability occurring across days that could influence the homeostatic response and to ensure a baseline level of corticomotor excitability that was consistent between individuals immediately prior to homeostatic plasticity induction, further 15 MEPs were recorded immediately prior to the induction of homeostatic plasticity (time point “baseline”) at an intensity sufficient to evoke an average MEP of 1 mV peak-to-peak amplitude (S_1mV_). This methodology is standard in studies of homeostatic plasticity [3, 4]. Homeostatic plasticity was induced in M1 using two blocks of anodal transcranial direct current stimulation (tDCS) applied for 7 min and 5 min, respectively and separated by a 3 min rest period (“double tDCS protocol”). This protocol has been used previously to induce homeostatic plasticity in human M1 [9, 14]. The corticomotor excitability in response to tDCS was monitored by recording 15 MEPs at S_1mV_ during the 3 min rest period between the two tDCS blocks (time point “between”), and at 0 min, 10 min, and 20 min follow-ups (see Figure 1). The number of MEPs was selected based on previous studies that have demonstrated good-to-excellent reliability when 15 MEPs are used to assess the corticomotor excitability within and between sessions [21–25].

### 2.3. Assessment of Corticomotor Excitability

Single-pulse transcranial magnetic stimulation (TMS) was delivered using a Magstim 200 stimulator (Magstim Co., Ltd., Dyfed, UK) and a standard 70 mm figure-of-eight coil. The coil was held over the left hemisphere, at a 45° angle to the sagittal plane to induce current in the posterior-anterior direction. The optimal coil position was determined by systematically moving the coil in 1 cm increments and locating the site that evoked the maximum response at the lowest stimulator intensity from the relaxed abductor pollicis brevis (APB) muscle (termed the “hotspot”). A soft-tip pen was used to mark the hotspot to allow accurate coil and tDCS electrode repositioning within and between testing sessions. Participants were requested to precisely remark their hotspot using a mirror and a soft-tipped pen or, if required, with assistance from a second person, on the days they did not attend the laboratory for testing. Surface electromyography was recorded using surface dual electrodes (Ag-AgCl, Noraxon dual electrodes, interelectrode distance: 2.0 cm) placed in a belly-tendon montage over the relaxed APB muscle [9, 11, 15]. The ground electrode was positioned over the ipsilateral olecranon. Raw EMG signals were amplified (1000 times), bandpass-filtered at 20–1000 Hz, and sampled at 2000 Hz (CED 1401 AD, Cambridge Electronic Design, Cambridge, United Kingdom) using Signal software (CED, version 5.08 × 86). To evaluate the change in the corticomotor excitability across days, 15 motor-evoked potentials (MEP) were recorded at 120% of resting motor threshold at the APB hotspot. The resting motor threshold (rMT) was defined as the minimum TMS intensity required to elicit at least five MEPs ≥ 50 *μ*V in ten consecutive trials from the resting APB muscle [26].

### 2.4. Induction and Monitoring of M1 Synaptic and Homeostatic Plasticity

A battery-driven, ramp-controlled, constant current stimulator (DC-Stimulator Plus, NeuroConn, Ilmenau, Germany) delivered two blocks of excitatory, anodal transcranial direct current stimulation (tDCS) to the left primary motor cortex (M1). The left M1 was targeted to control for hand dominance, as only right-handed individuals were included in this study. The first anodal tDCS block lasted for 7 min, and the second, for 5 min. The two blocks were separated by a 3 min rest period. Rubber electrodes, placed in NaCl-soaked sponges (5 × 7 cm) were positioned over the hotspot corresponding to the right APB muscle (anode) as determined above and over the contralateral supraorbital region (cathode). Electrodes were fixed in position with two adjustable rubber straps. The current intensity was ramped up (0 mA–1 mA) and down (1 mA–0 mA) over ten seconds at the start and end of stimulation [27]. The single-pulse TMS was used to monitor the corticomotor excitability in response to the first and second blocks of anodal tDCS. This was achieved by setting the stimulator intensity to S_1mV_ at the previously determined optimal scalp site.

### 2.5. Data Analysis

Data are presented as means and standard deviations (SD) in text, tables, and figures. Statistical analyses were conducted using SPSS software for windows, version 22.

The data distribution was assessed using the Shapiro-Wilk test. A one-way repeated measure ANOVA with the factor “day” (0, 2, 7, 14) was performed to compare (i) resting motor threshold, (ii) TMS intensity used to elicit S_1mV_, and (iii) corticomotor excitability (recorded at 120% rMT), between days. To examine the change in corticomotor response following the first block of anodal tDCS across days, the amplitude of the MEP at time point “between” was calculated as a proportion of the MEP amplitude at “baseline” and analysed using a one-way repeated measure ANOVA with the factor “day.” To examine the change in the corticomotor response to the double tDCS protocol across days, the amplitude of the MEP at each of the follow-up time points (0 min, 10 min, and 20 min) was calculated as a proportion of the MEP amplitude at time points “baseline” and “between,” and analysed using a one-way repeated measure ANOVA with the factor “day.” This analysis was performed as the magnitude of the homeostatic response is likely to be dependent on the corticomotor excitability at “baseline,” and the amount of facilitation achieved following the first block of anodal tDCS (i.e., time point “between”). Bonferroni post-hoc tests corrected for multiple comparisons were performed where appropriate. The Greenhouse-Geisser method was used to correct for nonsphericity. Effect sizes from the one-way repeated measure ANOVA are reported using partial eta squared. Cohen's benchmarks were used to define small (0.01), medium (0.06), and large effect sizes (0.14) [28, 29].

An intraclass correlation coefficient model (ICC 3,k) was used to evaluate the test-retest reliability of (i) the resting motor threshold, (ii) the TMS intensity used to elicit S_1mV_, (iii) the corticomotor excitability (recorded at 120% rMT), (iv) the corticomotor response to the first block of anodal tDCS, and (v) the corticomotor (homeostatic) response recorded at 0 min, 10 min, and 20 min after the second block of anodal tDCS, across days 0, 2, 7, and 14. The ICC 3,k model was used to determine consistency between variables across days by accounting for fixed effects from the rater and random effects from study participants [30, 31]. ICC scores ≤ 0.20 were considered poor: 0.2–0.40, fair: 0.41–0.60, moderate; 0.61–0.80, good; and ≥0.81, excellent [32].

## 3. Results

### 3.1. Corticomotor Excitability and Homeostatic Plasticity in Healthy Individuals at Intervals of 48 Hours, 7 Days, and 2 Weeks

All data had normal distribution. There was no difference in the resting motor threshold (*F*_2,16_ = 0.3, *P* = 0.7, partial eta squared = 0.03), the TMS intensity used to elicit S_1mV_ (*F*_3,27_ = 0.4, *P* = 0.7, partial eta squared = 0.04), or the corticomotor excitability (assessed at 120% rMT, *F*_2,16_ = 0.4, *P* = 0.6, partial eta squared = 0.05) between days (Table 1).

**Figure 2:**
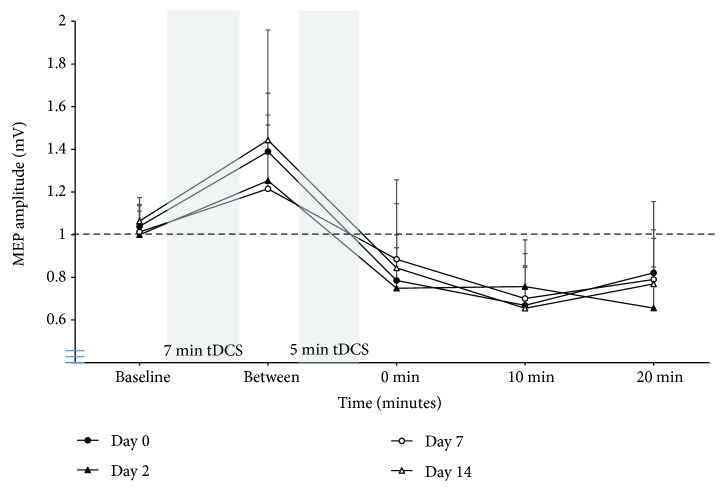
Group data (mean + SD) for motor-evoked potential (MEP) amplitude before the double tDCS protocol (“baseline”), after the first block of anodal tDCS (“between”), and at 0 min, 10 min, and 20 min follow-ups on days 0, 2, 7, and 14.

The magnitude of the increase in MEP amplitude following the first block of anodal tDCS was not different between days (corticomotor excitability at time point “between” calculated as a proportion of the MEP amplitude at “baseline”; *F*_3,27_ = 0.4, *P* = 0.8, partial eta squared = 0.04; Figure 2). Similarly, the magnitude of the decrease in MEP amplitude following the second block of anodal tDCS was not different between days at all follow-up time points (corticomotor excitability at time points 0, 10, and 20 min calculated as a proportion of the MEP amplitude at time point “baseline”; 0 min: *F*_2,16_ = 0.5, *P* = 0.5, partial eta squared = 0.06; 10-min: *F*_3,27_ = 1.7, *P* = 0.2, partial eta squared = 0.16; 20 min: *F*_3,27_ = 0.8, *P* = 0.5, partial eta squared = 0.08; and corticomotor excitability at time points 0, 10, and 20 min calculated as a proportion of the MEP amplitude at time point “between”; 0-min: *F*_3,27_ = 1.2, *P* = 0.3, partial eta squared = 0.12; 10 min: *F*_3,27_ = 1.3, *P* = 0.3, partial eta squared = 0.13; 20 min: *F*_3,27_ = 1.2, *P* = 0.3, partial eta squared = 0.12; Figure 2).

Small effect sizes were observed for rMT (partial eta squared = 0.03), the TMS intensity used to elicit S_1mV_ (partial eta squared = 0.04), the corticomotor excitability (assessed at 120% rMT, partial eta squared = 0.05), and the corticomotor response to the first block of anodal tDCS (partial eta squared = 0.04). Medium-to-large effect sizes were observed for homeostatic responses to the double tDCS protocol when normalised to “baseline” (0 min: partial eta squared = 0.06; 10 min: partial eta squared = 0.16; 20 min: partial eta squared = 0.08) and time point “between” (0 min: partial eta squared = 0.12; 10 min: partial eta squared = 0.13; 20 min: partial eta squared = 0.12).

### 3.2. Test-Retest Reliability

Excellent test-retest reliability was observed for rMT (ICC = 0.92, 95% CI 0.76–0.98; Table 1) and the TMS intensity used to elicit S_1mV_ (ICC = 0.95, 95% CI 0.87–0.99; Table 1) across days. Moderate-to-good reliability was observed for the corticomotor excitability assessed at 120% rMT across days (ICC = 0.80, 95% CI 0.47–0.94; Table 1).

The corticomotor response to the first block of anodal tDCS (ICC = 0.41, 95% CI −0.72–0.84; Table 1), and homeostatic responses to the double tDCS protocol at all follow-up time points across days, demonstrated moderate-to-good-reliability when data were normalised to time point “baseline” (0 min: ICC = 0.58, 95% CI −0.01–0.88; 10 min: ICC = 0.61, 95% CI −0.03–0.89; 20 min: ICC = 0.43, 95% CI −0.67–0.85; Table 1). Similarly, moderate-to-good-reliability was observed at all follow-up time points across days, when homeostatic responses were normalised to time point “between” (0 min: ICC = 0.61, 95% CI −0.03–0.89; 10 min: ICC = 0.67, 95% CI 0.12–0.91; 20 min: ICC = 0.60, 95% CI −0.06–0.89; Table 1). The highest ICCs were observed for the homeostatic plasticity response recorded at 10 min follow-up across days, (normalised to “baseline” ICC = 0.61, 95% CI −0.03–0.89; normalised to “between” ICC = 0.67, 95% CI 0.12–0.91; Table 1).

## 4. Discussion

This study is the first to examine the test-retest reliability of M1 homeostatic plasticity, induced and assessed using noninvasive brain stimulation, in the healthy human brain. The corticomotor response to single, and double, anodal tDCS demonstrated moderate-to-good test-retest reliability in healthy individuals over intervals up to 2 weeks. These data suggest that M1 homeostatic plasticity can be reliably induced and assessed over time using two blocks of anodal tDCS. This finding provides a foundation for the longitudinal evaluation of M1 homeostatic plasticity in humans using the double tDCS protocol.

Homeostatic plasticity regulates neuronal firing rates in the human brain and ensures that the neuronal activity is maintained within a stable physiological range [3, 4]. The Bienenstock-Cooper-Munro (BCM) theory of homeostatic plasticity proposes that neuronal firing rates are regulated based on the history of the postsynaptic activity, such that high levels of neuronal activity reduce the threshold for LTD induction and promote LTD-like plasticity (synaptic weakening, lower firing rates), while low levels of neuronal activity reduce the threshold for LTP induction and promote LTP-like plasticity (synaptic strengthening, higher firing rates) [2, 33].

Consistent with the BCM theory, studies exploring homeostatic plasticity using repetitive tetanic stimulation [5, 33–35] and noninvasive brain stimulation [3, 4, 36–38] have shown that neuronal activity is modified based on the level of postsynaptic activity [39–41]. For example, studies have shown that two blocks of anodal tDCS produce effects on M1 that follow a time-dependent rule consistent with homeostatic mechanisms [9]. Specifically, when 7 min of anodal tDCS is followed at 3 min interval by a second 5 min block of anodal tDCS, the increase in the corticomotor excitability observed with 7 min anodal tDCS applied alone is reversed toward inhibition [9]. The nature of this response mimics the homeostatic rule of a threshold that slides to favor the induction of LTD-like effects (the inhibitory response after the second block of anodal tDCS) when postsynaptic activity is high (following the first block of anodal tDCS) [2–4].

Our data confirm the direction and time course of these effects in the healthy brain (increased the corticomotor excitability in response to a single 7 min block of anodal tDCS; decreased the corticomotor excitability in response to double tDCS) and extend previous work by demonstrating moderate-to-good test-retest reliability with medium-to-large effect sizes when homeostatic plasticity is induced and assessed using noninvasive brain stimulation at intervals of 48 hours, 7 days, and 2 weeks. Specifically, moderate-to-good test-retest reliability with medium-to-large effect sizes was observed when the magnitude of the homeostatic response was considered relative to “baseline,” (all ICC ≥ 0.43; all partial eta squared ≥ 0.06; Table 1) and when the magnitude of the response was considered relative to the level of facilitation produced following the first block of anodal tDCS (all ICC ≥ 0.60; all partial eta squared ≥ 0.12; Table 1). The greatest test-retest reliability (ICC ≥ 0.61) with the largest effect size (partial eta squared ≥ 0.13) was observed when the homeostatic response was evaluated at the 10 min follow-up.

The current data also provide further evidence that the resting motor threshold (ICC = 0.92, 95% CI 0.76–0.98) and the corticomotor excitability (ICC = 0.80, 95% CI 0.47–0.94) are reliable at intervals of 48 hours, 7 days, and 2 weeks. This finding is in agreement with previous studies. For example, Malcolm et al. (2006) reported high reliability in motor thresholds (ICC = 0.90–0.97) in healthy volunteers over a period of 2 weeks [42]. Further, good reliability (ICC ≥ 0.75) for cortical excitability measures (resting motor threshold, TMS input-output curves, MEP amplitude, and cortical silent period) have been reported across two testing sessions, each 1 week apart, in healthy volunteers [43]. As changes in the resting motor threshold and/or baseline corticomotor excitability are likely to influence the homeostatic response, the reliability of these measures over time is an important consideration in the assessment of homeostatic plasticity in humans [3, 12, 44].

Previous studies have used a range of noninvasive brain stimulation protocols to probe the M1 homeostatic plasticity in both healthy and clinical populations [3, 4, 11, 13]. In people with nonspecific chronic low back pain (cLBP), homeostatic plasticity was assessed in M1 using a double tDCS protocol identical to that investigated here [14]. The authors demonstrated the impaired homeostatic plasticity in this population characterised by a failure to reverse high corticomotor excitability (induced by the first block of tDCS) toward inhibition (following the second block of tDCS). Using 5 Hz trains of repetitive TMS, the impaired homeostatic plasticity has been reported in individuals with episodic migraine during the preictal and postictal stages of the migraine cycle [12]. Although data were obtained from different individuals at different stages of the migraine cycle (i.e., the study did not utilise a repeated-measures design), impaired homeostatic plasticity was theorised to contribute to headache recurrence and migraine transformation from an episodic to a chronic condition [12]. Similar observations were reported in the M1 of individuals with focal hand dystonia where patients failed to reverse high corticomotor excitability toward inhibition when 1 Hz rTMS was primed by anodal tDCS [15]. Impaired M1 homeostatic plasticity in focal hand dystonia was later reported to correlate with the severity of symptoms and hypothesised to contribute to aberrant sensorimotor plasticity in this condition [13]. These data have been interpreted to suggest that impaired homeostatic plasticity may play a role in the pathogenesis of some clinical conditions. Further exploration of these findings using longitudinal and repeated measures study designs are needed to confirm these hypotheses.

It is noteworthy that some studies using repeated noninvasive brain stimulation techniques have demonstrated nonhomeostatic interactions in the human M1, where cumulative (rather than opposite) LTP- or LTD-like effects are induced [3, 45, 46]. For example, the application of two successive inhibitory continuous theta-burst stimulation protocols results in long-lasting MEP depression and not a reversal toward facilitation as would be hypothesised by the BCM theory [47, 48]. These data suggest that in addition to homeostatic mechanisms, nonhomeostatic interactions might also shape noninvasive brain stimulation-induced LTP-like and LTD-like effects. Future studies exploring the interplay between homeostatic and nonhomeostatic mechanisms over time are warranted in healthy and pathological populations.

This study has several limitations. First, the test-retest reliability in M1 homeostatic plasticity was assessed in one direction only, that is, with a facilitatory priming protocol (anodal tDCS). This approach was selected as previous studies in pathological conditions have shown failure to induce LTD when postsynaptic activity is high [11, 13]. However, since the polarity and magnitude of synaptic plasticity varies as a function of activation history in the postsynaptic neuron, future studies should seek to determine whether inhibitory priming protocols (e.g., cathodal tDCS) are also reliable over time. Second, this study did not assess homeostatic plasticity in intracortical inhibitory or facilitatory networks. As tDCS is known to influence intracortical activity [27, 49–51], and homeostatic impairment has been demonstrated in intracortical inhibitory and facilitatory networks in individuals with migraine [16, 17, 44, 52], future studies should investigate homeostatic regulation in these networks over time. Third, although this study used noninvasive brain stimulation methods similar to previous studies in this field [3, 4], tDCS applied to M1 using electrodes of 5 × 7 cm^2^ may have resulted in the current spread to surrounding cortical regions [27, 53, 54]. Finally, our findings are limited to homeostatic plasticity in the healthy M1 using a double tDCS protocol. Further research is needed to determine the test-retest reliability of homeostatic plasticity induced using other noninvasive brain stimulation methodologies in M1, as well as homeostatic plasticity induced in other brain regions relevant to different pathologies [55–58].

## 5. Conclusion

These data demonstrate that M1 homeostatic plasticity, induced using two blocks of anodal tDCS and assessed using single-pulse TMS, has moderate-to-good reliability at intervals of 48 hours, 7 days, and 2 weeks, with the greatest reliability observed when the homeostatic response is assessed at the 10 min follow-up. These findings provide a foundation for the assessment of homeostatic plasticity in the primary motor cortex using repeated measures and longitudinal study designs in humans.

## Figures and Tables

**Table 1:** Test-retest reliability (intraclass correlation coefficient [ICC]) estimates for cortical measures recorded across days 0, 2, 7, and 14.

Cortical measures	Cortical measures across days				ICC (95% CI)
	Day 0 (mean + SD)	Day 2 (mean + SD)	Day 7 (mean + SD)	Day 14 (mean + SD)	
rMT (% maximum stimulator output)	44 ± 7	45 ± 6	45 ± 7	44 ± 6	0.92 (0.76–0.98)
S_1mV_ (% maximum stimulator output)	54 ± 9	55 ± 11	56 ± 12	55 ± 12	0.95 (0.87–0.99)
Corticomotor excitability (mV)	1.0 ± 0.5	1.2 ± 0.9	1.0 ± 0.8	1.1 ± 0.9	0.80 (0.47–0.94)
Corticomotor response_baseline_ (mV)	1.4 ± 0.3	1.3 ± 0.3	1.2 ± 0.3	1.4 ± 0.5	0.41 (−0.72–0.84)
Homeostatic response_baseline_ 0 min (mV)	0.8 ± 0.2	0.8 ± 0.2	0.8 ± 0.4	0.8 ± 0.3	0.58 (−0.01–0.88)
Homeostatic response_baseline_ 10 min (mV)	0.7 ± 0.2	0.8 ± 0.2	0.7 ± 0.2	0.6 ± 0.1	0.61 (−0.03–0.89)
Homeostatic response_baseline_ 20 min (mV)	0.8 ± 0.4	0.7 ± 0.2	0.8 ± 0.2	0.7 ± 0.2	0.43 (−0.67–0.85)
Homeostatic response_between_ 0 min (mV)	0.6 ± 0.1	0.6 ± 0.2	0.8 ± 0.4	0.7 ± 0.4	0.61 (−0.03–0.89)
Homeostatic response_between_ 10 min (mV)	0.5 ± 0.2	0.6 ± 0.2	0.6 ± 0.3	0.5 ± 0.2	0.67 (0.12–0.91)
Homeostatic response_between_ 20 min (mV)	0.6 ± 0.3	0.5 ± 0.2	0.7 ± 0.3	0.6 ± 0.2	0.60 (−0.06–0.89)

Cortical measures: (i) resting motor threshold (rMT), (ii) transcranial magnetic stimulator (TMS) intensity needed to elicit an average peak-to-peak MEP amplitude of 1 mV(S_1mV_), (iii) corticomotor excitability (motor-evoked potential (MEP) amplitude recorded at 120% of rMT), (iv) the corticomotor response to the first block of anodal tDCS normalised to baseline (corticomotor response_baseline_), and (v) the corticomotor (homeostatic) response to the second block of anodal tDCS normalised to “baseline” (homeostatic response_baseline_) and “between” (homeostatic response_between_) at 0 min, 10 min, and 20 min follow-ups.
